# Accreditation as a driver of interprofessional education: the Canadian experience

**DOI:** 10.1186/s12960-022-00759-4

**Published:** 2022-08-26

**Authors:** Mohammad B. Azzam, Marie-Andrée Girard, Cynthia Andrews, Hope Bilinski, Denise M. Connelly, John H. V. Gilbert, Christie Newton, Ruby E. Grymonpre

**Affiliations:** 1 Curriculum Studies and Studies in Applied Linguistics, Faculty of Education, Western University, London, ON Canada; 2 Anesthesiology and Pain Medicine Department, Faculty of Medicine, University of Montreal, Montreal, QC Canada; 3 Health Hub: Politics, Organizations and Law, Montreal, QC Canada; 4 Department of Dental Clinical Sciences, Faculty of Dentistry, Dalhousie University, Halifax, NS Canada; 5 College of Nursing, University of Saskatchewan, Saskatoon, SK Canada; 6 School of Physical Therapy, Faculty of Health Sciences, Western University, London, ON Canada; 7 College of Health Disciplines, University of British Columbia, Vancouver, BC Canada; 8 Department of Family Practice, Faculty of Medicine, University of British Columbia, Vancouver, BC Canada; 9 College of Pharmacy, Rady Faculty of Health Sciences, University of Manitoba, Winnipeg, MB Canada

**Keywords:** Interprofessional education, Health and social care professions, Accreditation, Canada

## Abstract

**Background:**

The purpose of this study was to (1) explore evidence provided by Canadian health and social care (HASC) academic programs in meeting their profession-specific interprofessional education (IPE)-relevant accreditation standards; (2) share successes, exemplars, and challenges experienced by HASC academic programs in meeting their IPE-relevant accreditation standards; and (3) articulate the impacts of IPE-relevant accreditation standards on enabling interprofessional learning to the global HASC academic community.

**Methods:**

Profession-specific (bilingual, if requested) surveys were developed and emailed to the Deans/Academic Program Directors of eligible academic programs with a request to forward to the individual who oversees IPE accreditation. Responses were collated collectively and by profession. Open-ended responses associated with our first objective were deductively categorized to align with the five Accreditation of Interprofessional Health Education (AIPHE) standards domains. Responses to our additional questions associated with our second and third objectives were inductively categorized into themes.

**Results/discussion:**

Of the 270 HASC academic programs surveyed, 30% (*n* = 24) partially or completely responded to our questions. Of the 106 IPE-relevant standards where evidence was provided, 62% (*n* = 66) focused on the *Educational Program*, 88% of which (*n* = 58) were either met or partially met, and 47% (*n* = 31) of which focused on practice-based IPE. Respondents cited various exemplars and challenges in meeting IPE-relevant standards.

**Conclusions:**

The overall sentiment was that IPE accreditation was a significant driver of the IPE curriculum and its continuous improvement. The array of exemplars described in this paper may be of relevance in advancing IPE implementation and accreditation across Canada and perhaps, more importantly, in countries where these processes are yet emerging.

**Supplementary Information:**

The online version contains supplementary material available at 10.1186/s12960-022-00759-4.

## Background

The World Health Organization (WHO, [1–3]) has increasingly emphasized that the world is facing a workforce crisis in health and social care (HASC), with a projected worldwide shortage of 18 million HASC providers by 2030 [4]. To address this shortage, the WHO published its *Health in all Policies* (HiAP) document [5] that calls for systemic, long-term, cross-sectoral policy coherence to promote new ways of working in the HASC sector. As underscored by the Lancet report [6] and in the WHO’s Module 3-02 of the National Health *Workforce Accounts* (NHWA) [7], accreditation plays a significant role in influencing HASC professional education and should be considered a global theme that is addressed by all HASC systems so that HASC reform aligns with social accountability. Further, in WHO’s *Framework for Action on Interprofessional Education and Collaborative Practice* [2], Action 2 emphasizes the need to “Create accreditation standards for [HASC] worker education programmes that include clear evidence of interprofessional education” (p. 10). The need to embed IPE in accreditation standards is also noted in Standard 3-06 of the NHWA [7], which states that the “existence of national and/or subnational standards for interprofessional education in accreditation” are necessary to inform HASC workforce policy and practices (p. 45).

Canada has the longest standing collective experience developing IPE-relevant accreditation standards. Between 2007 and 2011, the Health Canada-funded Accreditation of Interprofessional Health Education (AIPHE) projects [8, 9] engaged eight Canadian accreditation agencies of six HASC professions to develop accreditation standards for IPE [10]. Phase 1 resulted in a set of guiding principles for IPE standards [8]. Phase 2 engaged a broader group of key stakeholders including regulators, professional associations, clinician managers, government, and educators to arrive at a consensus on IPE standards’ language and examples of evidence [9]. Through this process, AIPHE participants agreed to frame IPE-relevant accreditation standards around five *domains*: *Organizational Commitment*, *Faculty*, *Students*, *Educational Program*, and *Resources* ([9]; Table 1).

**Table 1:** The accreditation standards domains identified in the AIPHE project [8]. Extracted verbatim, with permission, from [11]

Domain	Description
Organizational commitment	Organizational commitment refers to administrative structures and processes, preferably at the level of the Vice President’s Office and/or deanship, that must foster the development, implementation, and evaluation of interprofessional education
Faculty	Faculty members must be supported, encouraged, and prepared to facilitate the development, implementation, and evaluation of interprofessional education
Students	Students must understand the significance of interprofessional education and demonstrate proficiency in interprofessional competencies
Educational program	Educational programs within and across faculties must share a common understanding of IPE and facilitate the development, implementation, and evaluation of interprofessional education throughout the learning continuum for all students
Resources	The human, material, and financial resources that enable the development, implementation, and evaluation of interprofessional education must be supplied

*AIPHE* Accreditation of Interprofessional Health Education

Recently, a comparative content analysis examined IPE language within the accountable^1^ statements in 13 accreditation standards documents for 11 Canadian HASC professions [11], six of which were involved in the AIPHE projects [8, 9], suggesting that implementation of the IPE language in the standards was greater than the AIPHE projects. It was encouraging to see frequent reference to IPE, with 92% (*n* = 12) of the 13 accreditation standards documents specifying 77 IPE-relevant accountable statements (for an explication of the IPE-relevant text for each profession, see Additional file 1). These findings suggest a positive impact of the Canadian AIPHE projects on HASC academic programs.

With close to a decade of experience during which accrediting organizations have collectively been seeking evidence of IPE within their respective HASC academic programs, Canada is well positioned to examine the downstream effects of IPE accreditation on academic structures, processes, and HASC professional program delivery. In response, the Canadian Interprofessional Health Collaborative (CIHC) Accreditation Working Group conducted a survey to answer the following three research questions: (1) What form of evidence is being provided by academic programs that participated in the 2007–2011 AIPHE projects to meet current profession-specific IPE-relevant accreditation standards? (2) Were there IPE-relevant accreditation standards and was evidence provided by those eligible HASC professions that did not participate in the AIPHE projects? (3) What are the successes and challenges reported by participating academic programs in implementing strategies to address IPE-relevant accreditation standards?

## Methods

As this study involved collection and analysis of organizational level data, the requirement for ethics approval was waived by the Health Research Ethics Board (HREB) at both the University of Montreal and University of Manitoba.

### Identifying eligible HASC professions

**Fig. 1:**
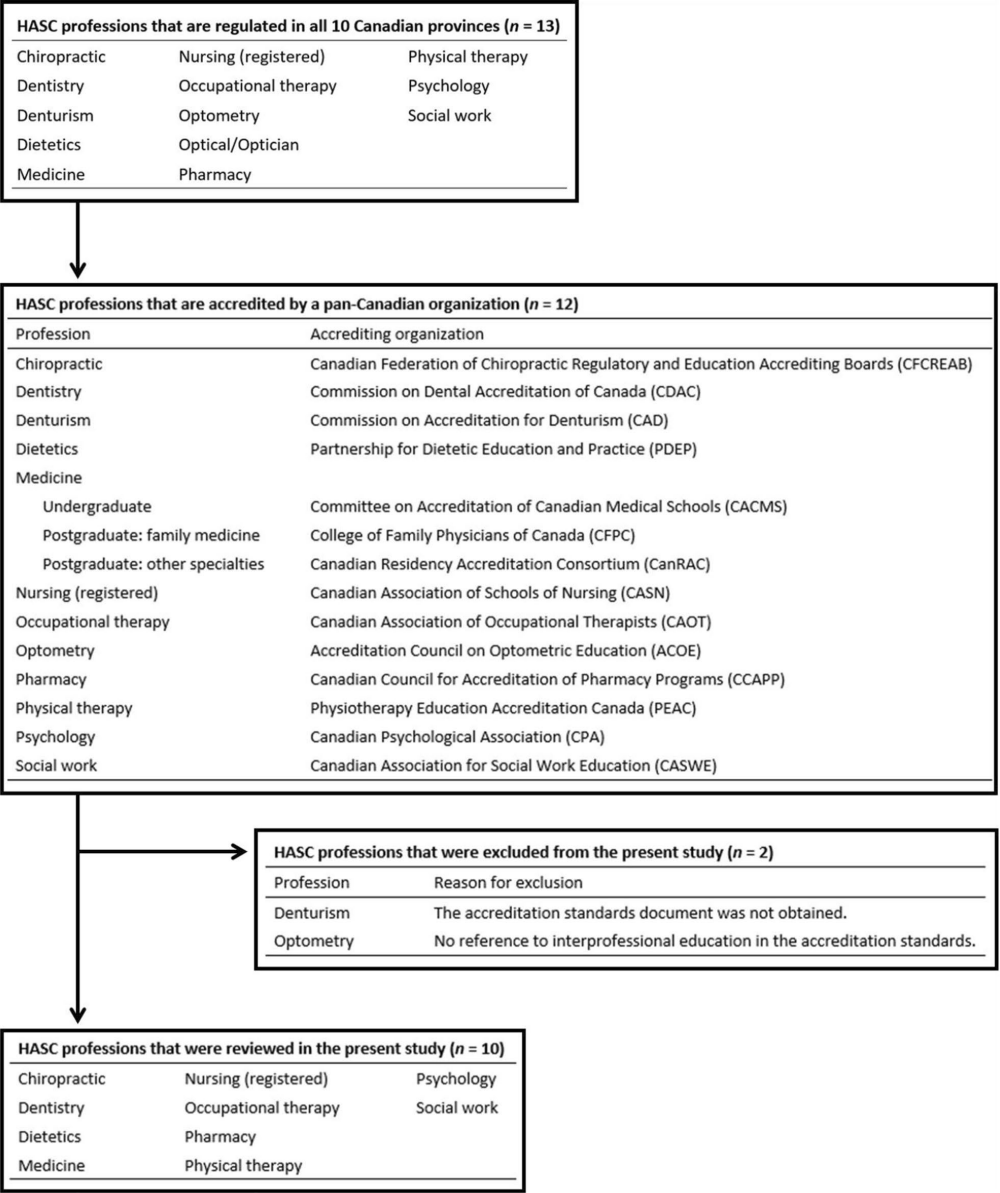
Inclusion and exclusion of the 13 regulated health and social care (HASC) professions and their accrediting organizations

HASC professions in Canada were eligible for this study if: (1) they were regulated by provincial legislation and by a specific provincial regulatory body in all 10 Canadian provinces^2^ and (2) the academic programs that lead to professional licensure in every province were accredited through a single pan-Canadian organization (Fig. 1). Given these eligibility criteria, only 12 professions were therefore eligible to participate in this study: chiropractic, dentistry, denturism, dietetics, medicine, nursing (registered), occupational therapy, optometry, pharmacy, physical therapy, psychology, and social work.

In preparation for survey development, the accreditation standards documents for these professions were retrieved and reviewed; the Commission on Accreditation for Denturism (CAD) did not respond to our request for their accreditation standards document; therefore, denturism was excluded from this study. Similarly, optometry was further excluded after review of the Accreditation Council on Optometric Education (ACOE)’s accreditation standards document [12] revealed that their accreditation standards made no reference to IPE or IPCP.

Table 2 lists the 16 versions of accreditation standards [13–28] used for survey development for the 10 eligible HASC professions. The number of accreditation standards documents retrieved and reviewed exceeded the number of eligible HASC professions as medical education (and its respective accreditation processes) involves undergraduate education followed by residency in either family medicine or other specialty medicine. Thus, these three divisions of medical education were considered separately as each category was accredited by a different organization. Further, if the publication date of the most recent version of a profession’s accreditation standards document was 2017 or later, the previous version of standards for that profession was also retrieved, if available. This was performed to increase the response rate as, depending on each profession’s accreditation review cycle, many academic units would have been excluded from this study if their most recent accreditation review were prior to 2017 and assessed against the older version of standards. This was the case for three professions (undergraduate medicine, occupational therapy, and pharmacy); postgraduate medical programs are typically accredited simultaneously and were assessed against the 2018 accreditation standards; therefore, retrieval of earlier versions was not necessary.

**Table 2:** Versions of the accreditation standards documents used for survey development

Profession	Accrediting organization	Version
Chiropractic	Canadian Federation of Chiropractic Regulatory and Education Accrediting Boards (CFCREAB)	2011 [13]
Dentistry	Commission on Dental Accreditation of Canada (CDAC)	2013 [14]
Dietetics	Partnership for Dietetic Education and Practice (PDEP)	2014 [15]
Medicine		
Undergraduate	Committee on Accreditation of Canadian Medical Schools (CACMS)	2015 [16], 2019 [17]
Postgraduate: family medicine	College of Family Physicians of Canada (CFPC)	2018 [18]
Postgraduate: other specialties	Canadian Residency Accreditation Consortium (CanRAC)	2018 [19]
Nursing (registered nurse)	Canadian Association of Schools of Nursing (CASN)	2014 [20]
Occupational therapy	Canadian Association of Occupational Therapists (CAOT)	2011 [21], 2017 [22], 2019 [23]
Pharmacy	Canadian Council for Accreditation of Pharmacy Programs (CCAPP)	2013 [24], 2018 [25]
Physical therapy	Physiotherapy Education Accreditation Canada (PEAC)	2012 [26]
Psychology	Canadian Psychological Association (CPA)	2011 [27]
Social work	Canadian Association for Social Work Education (CASWE)	2014 [28]

### Creating and distributing the surveys

The surveys were tailored to each HASC profession’s accreditation standards (for an explication of the IPE-relevant text for each profession, see Additional file 1). All accountable IPE-relevant text was constructed into survey questions guided by a template. The greater the number of IPE-relevant text cited in the standards, the greater the number of survey questions. For example, in the Canadian Association of Schools of Nursing (CASN, [20]) accreditation standards document, IPE-relevant criteria were explicitly stated within five *pillars*: Partnerships, Teaching and Learning, Environment, Program Framework, and Professional Growth. This required constructing five survey questions, one for each of the five pillars, using the following template: “What types of evidence did your Academic Program provide to fulfill the IPE-relevant components of the Pillar: X? Feel free to either type in or cut and paste from any document into this comment box.”

Respondents were also asked to respond to three additional questions:

1. For those IPE-relevant standards that were *met* by your academic program can you describe, in greater detail, one or two exemplars/innovative approaches?
2. For those IPE-relevant standards that were *partially met* or *not met* by your academic program, what are reported challenges?
3. Can you share with us your general perceptions/reflections regarding the impact of IPE-relevant accreditation standards on enabling/fostering interprofessional learning within your academic program?

The surveys for each profession were uploaded onto the web-based platform, SurveyMonkey™. An invitation email was sent to all Deans/Academic Program Directors of the relevant Schools, Colleges, and Faculties with the instructions to forward the survey link and consent to the individual in charge of accreditation for their academic programs. When standards were available in French and/or by request, a French version of the survey was distributed to French language institutions. Reminders to complete the survey were sent every 2 weeks over a 6-week period between October and December 2020.

### Response metrics of web-based surveys

Compared to traditional, paper-based surveys, there is a greater likelihood that respondents of web-based surveys are non-representative and/or biased through a self-selection (volunteer) effect [29]. Further, response rates of paper-based surveys are straightforward, whereby the response rate is typically calculated by dividing the number of respondents by the number of surveys sent. Conversely, web-based surveys typically require multiple layers of responses and sequential numerators and denominators. For this reason, Eysenbach advises against the use of the term *response rate* and recommends clearly defined *response metrics*. Consistent with Eysenbach’s recommendations, the response metrics for our survey included five parameters (Table 3).

**Table 3:** Response metrics, consistent with Eysenbach’s [29] recommendations

Metric	Definition
Unique visitor	The number of unique visitors to the web-based survey. We tracked and noted the IP address for each responder to avoid multiple answers from the same respondent
View rate	The ratio of the number of unique visitors to the first page of the survey to the number of unique site visitors. It is not unusual to have view rates < 0.1% for a voluntary survey
Participation rate or recruitment rate	The ratio of the number of unique visitors who completed the first page of the survey (e.g., checked a check box) to the number of unique visitors to the first page of the survey
Completion rate	The ratio of the number of unique visitors who completed the survey (e.g., submitted the last page of the survey) to the number of unique visitors who completed the first page of the survey. Note that this is a measure of attrition, not a measure of completeness
Completeness rate	The ratio of the number of unique visitors who completed all items to the number of unique visitors who completed the first page of the survey

### Data analysis

Response metrics were calculated according to the five parameters listed in Table 3 for each profession separately. All responses were analyzed, regardless of the proportion of survey items answered. All profession-specific, IPE-relevant data were deductively categorized into the five accreditation standards domains identified by AIPHE ([9]; Table 1); whereas, the responses to the additional three questions (exemplars/innovative approaches, challenges, and impact of IPE-relevant accreditation standards) were inductively categorized into themes.

## Results

Ten HASC professions met the eligibility criteria for this study (Fig. 1). Analyses of the surveyed data, however, revealed that at least 16 different HASC professions collectively engaged in IPE activities. In addition to the surveyed 10 professions, the other participating HASC professions reportedly were audiology, dental hygiene, healthcare aides, physician assistant, respiratory therapy, and speech-language pathology. Additionally, of the 270 HASC academic programs surveyed, 80 visited the survey site (30% view rate), 24 of which responded at least partially to the survey (30% participation rate). Only 12 of these 24 programs completed all survey questions (50% completeness rate; Table 4).

**Table 4:** Response metrics, consistent with Eysenbach’s [35] recommendations

Profession	Programs surveyed (*N*)	Unique visitors (*n*)	No responses (*n*)	Partial responses (*n*)	Complete responses (*n*)	View rate (%)	Participation rate (%)	Completion rate (%)	Completeness rate (%)
Chiropractic	3	3	3	0	0	100	0	N/A	N/A
Dentistry	10	8	2	3	3	80.0	75.0	100	50.0
Dietetics	18	7	5	1	1	38.9	28.6	100	50.0
Medicine									
Undergraduate	17	10	9	0	1	58.8	10.0	100	100
Postgraduate: family medicine	17	2	2	0	0	11.8	0	N/A	N/A
Postgraduate: other specialties	17	8	8	0	0	47.1	0	N/A	N/A
Nursing (registered nurse)	53	11	6	1	4	20.8	45.5	100	80.0
Occupational therapy	11	4	2	1	1	36.4	50.0	100	50.0
Pharmacy	19	9	6	3	0	47.4	33.3	100	0
Physical therapy	15	10	6	2	2	66.7	40.0	100	50.0
Psychology	50	4	4	0	0	8.0	0	N/A	N/A
Social work	40	4	3	1	0	10.0	25.0	100	0
Total	270	80	56	12	12	29.6	30.0	100	50.0

Further, Table 5 outlines the frequency with which IPE-relevant accreditation standards were reportedly *met*, *partially met*, or *not met* by the respondents. Of the 106 IPE-relevant standards where evidence was provided, 66 (62%) focused on the *Educational Program*. Fifty-eight (88%) of these 66 *Educational Program* standards were either met or partially met. Of particular significance was that 31 (47%) of these 66 *Educational Program* IPE-relevant standards focused on practice-based^3^ education, and 27 (87%) of these 31 practice-based IPE standards was either met or partially met.

**Table 5:** Status of IPE-relevant standards, by accreditation standards domain [9]

Accreditation standards domain	Met	Partially met	Not met	Do not know	Total
Organizational commitment	9	3	0	1	13
Relationships	3	0	0	3	6
Endorsement	5	1	0	1	7
Faculty	9	0	0	3	12
Students					
Educational program	26	5	1	3	35
Didactic	25	2	1	3	31
Practice-based	2	0	0	0	2
Resources					
Total	79	11	2	14	106

### Exemplars/innovative approaches

Respondents from the HASC academic programs cited various exemplars as evidence in meeting IPE-relevant standards which aligned with the five AIPHE accreditation standards domains ([9]; Table 1). The greatest number of IPE exemplars addressed the *Educational Program* domain. Exemplars included facilitated, small group, case-based and/or problem-based interprofessional learning with some programs using simulation and standardized patients. An unexpected finding was the high number of practice-based interprofessional learning opportunities reported including interprofessional screening clinics, interprofessional hospital-based internships/placements in geriatrics and pediatrics, and interprofessional immersion fieldwork placements in First Nations communities. Some academic programs have also implemented mandatory IPE courses. One noteworthy example was a mandatory longitudinal IPE curriculum with students assigned to the same interprofessional team over a 2-year time period. The longitudinal curriculum was described as utilizing various educational approaches, including asynchronous online activities, facilitated face-to-face small group discussions, individual reflections, and a second-year capstone interprofessional team assignment.

IPE exemplars used as evidence to address the *Organizational Commitment* domain included the establishment of an Office of Interprofessional Education/Collaboration or a university-wide advisory/steering committee or network, development of an organizational structure, delegating IPE/IPCP to a senior administrator (such as a Vice-Dean), appointing faculty champions as didactic and practice-based IPE coordinators, and endorsing contractual agreements. Organizational commitment was also demonstrated through relationship building and partnerships among academic programs from a reported 16 different HASC professions. Formalizing partnerships with other post-secondary education institutions (including technical vocational schools) was particularly noteworthy as was partnership with patients on committees and as student mentors.

Few IPE exemplars addressed the *Resources*, *Students*, and *Faculty* accreditation standards domains [9]. Evidence of dedicating resources towards IPE included endorsed written agreements to IPE, faculty time and salary allotment for IPE, and funding to support interprofessional research and scholarship. Noteworthy *Students*-relevant exemplars included a student-inspired Health Sciences Students Association (HSSA) to promote interprofessional opportunities and relationships. *Faculty*-relevant exemplars development in IPE included training courses, such as the *Educating Health Professionals in Interprofessional Care* (EHPIC™) course, and interprofessional continuing professional development. Another faculty-related exemplar identified patient engagement and their facilitation of IPE activities. One respondent stated that,

Students are required to participate, as members of multidisciplinary teams of four students. [In this program], the “patients” are the student mentors (a.k.a. “teachers”). The mentors are people who have endured and possibly suffered from chronic conditions for significant periods of time in their lives. The objective of the [program] is to facilitate students, who will become [HASC] professionals, learning and appreciating of the meaning and life impact of living with a significant chronic condition.

### Impacts of IPE accreditation

IPE accreditation standards were noted to be a significant driver of interprofessional curriculum content and its continuous improvement. One respondent stated that the IPE-relevant accreditation standards serve as a “huge enabler! Accreditation drives curriculum content.” Further, the standards helped justify the provision of resources to meet those standards and inform the programs’ strategic plans.

### Challenges

The challenges cited by the respondents were categorized into four main themes: (1) poor student engagement; (2) language difficulties; (3) scheduling; and (4) inadequate accreditation standards. The most cited challenge was associated with poor student engagement, mainly due to participatory discrepancies across the HASC professions, where students of some professions were obliged to participate while participation for others was non-mandatory. One respondent indicated that,

Other faculties do see the benefits [in participating] and this is why they participate with us but if it is not required, the commitment is not present in the same way for all. We are on different pages for level of importance.

Further, another challenge was associated with the language barriers experienced between French and English language faculties and/or academic institutions that have interprofessional relationships. One respondent asserted that they realize programs offered in French have “limited access to other [francophone HASC] programs.”

Scheduling interprofessional learning opportunities between and across professions was another noted challenge. Some respondents also noted a lack of clarity regarding IPE-relevant accreditation standards in their respective accreditation standards documents and the nature of evidence being requested. While one respondent contended that “I wish the standards were more explicit related to IPE”, another respondent verified that “We don't always know how to respond to them with what we can do in our current situation.”

## Discussion

As one of the first bilingual studies on the subject, this study allowed perspectives to be heard from more inclusive and diverse communities across Canada. Further, this is the first published study to explore how HASC academic programs are meeting their profession-specific IPE accreditation standards and to illustrate the impacts of accreditation on the Canadian IPE experience. Thus, our findings are not meant to be generalizable. Rather, our hope is that the themes that emerged from this study have relevance to the global HASC academic community where IPE may be less developed.

Given the qualitative nature of this study, the rich quality of responses is more invaluable compared to the low response rate. It was encouraging to see the array of evidence provided to demonstrate innovative and theoretically grounded interprofessional learning opportunities offered within and among pre-licensure HASC professional programs. It was also promising that respondents believed that accreditation serves as an enabler of IPE implementation. This position was also seen in a recent Australian–Dutch study by Akdemir et al. [30], who examined the impacts of accreditation in practice on postgraduate medical programs from the perspectives of accreditors, clinicians, and trainees. Akdemir et al. stated that,

All participants acknowledged the necessity of accreditation to evaluate quality of training, despite its substantial costs, time-consuming nature, and emotional burden. Many participants argued that without standards it would be difficult to assure a minimum level of quality. […] Trainees mentioned the need for an impartial and objective perspective on training quality by an accreditation authority. In addition, supervisors find accreditation reports useful to demand changes or resources from the hospital administration. (p. 3)

IPE is defined by the Centre for the Advancement of Interprofessional Education (CAIPE) as “occasions when members or students of two or more [HASC] professions learn with, from and about each other to improve collaboration and the quality of care and services” ([31], p. 1). By definition, IPE is therefore an educational strategy that requires interaction and active engagement among learners from different HASC professions. That being said, current IPE-relevant accreditation standards only capture *occasions* for IPE, but do not capture evidence of the quality of these interprofessional learning opportunities. As such, this study could not assess the quality of implemented IPE activities; consequently, data may not have been readily made available to us. Further, the AIPHE projects [8] emphasized that,

Something must be exchanged among and between learners from different professions that changes how they perceive themselves and others. These changes must positively affect clinical practice in a way that enhances interprofessional collaboration, client involvement in care, and ultimately improves health outcomes. (p. 8)

Subsequently, the reported exemplars involving interprofessional practice-based learning are particularly noteworthy. Moving forward and to show the impacts of IPE activities on the achievement of IPE competency domains by program graduates leading to IPCP, there is a need for IPE-relevant accreditation standards to request evidence of the quality of IPE application in both didactic and practice-based settings. One study suggests that clinician team facilitation and mentorship of senior pre-licensure learners participating in interprofessional clinical placements lead to greater clinician personal awareness of interprofessional teaming, reflection, and changes in their own practice and mentorship of students [32]. In parallel with the growing IPE movement, the hope is that health services delivery accreditation standards further catalyze IPCP. It is encouraging that the Canadian Health Services Organization (HSO), responsible for developing protocols for accreditation of health services delivery, has recently written *Clinical Governance Standards* [33] to guide clinical management and service providers. A major theme throughout the guide is to ensure that,

Everyone in and associated with the organization (leaders, providers, patients/clients, families including caregivers, community members and partners in the system) work collaboratively in a team-based and interprofessional manner to provide clients with the right care at the right time for the best possible client experience and outcomes.

Additionally, it is somewhat concerning that the focus of IPE accreditation standards evidence is on the *Educational Program* domain with a much lower emphasis placed on other accreditation standards domains (*Organizational Commitment*, *Faculty*, *Students*, and *Resources*). These findings are consistent with the Canadian review of IPE-relevant accountable statements which reported emphasis primarily on the *Students* and *Educational Program* domains [11]. At both the macro-level and meso-level, the D’Amour framework [34] suggests that IPE program sustainability is threatened by a lack of a collective vision and sincere organizational commitment to IPE. Additionally, non-supportive administrative processes, including siloed resources and tenure and promotion criteria that do not reward IPE pose challenges to sustainability. At the micro-level, lack of faculty development in IPE and inclusion of patients as facilitators of IPE [35], limited student engagement, and learning contexts not grounded in adult learning theories stifle innovation and threaten program quality [36].

Most of the challenges cited by the respondents are not surprising and have been reported previously [37]. The use of an adoption model framework, such as the D’Amour framework [34], by program planners would facilitate diffusion of an innovation such as IPE within and between organizations and sectors, and prospectively identify and address anticipated challenges [36]. The lack of mandatory student engagement and/or varying student/faculty perceptions regarding the importance of IPE noted as challenges in this study are micro-level education and socialization factors identified in the D’Amour framework. Similarly, the challenges of scheduling and collaboration among other (francophone) academic, vocational, or technical institutions and practice environments reported in this study can be anticipated as meso-level, institutional factors (leadership, resources, administrative processes) within the D’Amour framework.

Accreditation of IPE was viewed very positively by survey respondents with a sentiment that this external review process facilitates resource allocation to support IPE innovation, drives program implementation, and promotes ongoing program reflection and improvement. The inclusion of accountable IPE-relevant language in the accreditation standards for 10 HASC professions and the reported engagement of over 16 different HASC professions in IPE exemplars suggests that the AIPHE projects [8, 9], involving six of these HASC professions (medicine, nursing, pharmacy, physical therapy, occupational therapy, and social work), had significant influence on those academic programs not involved in the AIPHE projects (e.g., chiropractic, dentistry, dietetics, and psychology) as well as those HASC professions that reportedly participate in IPE activities but are neither regulated in all 10 Canadian provinces nor accredited by a pan-Canadian organization (e.g., audiology, dental hygiene, healthcare aides, physician assistant, respiratory therapy, and speech-language pathology).

## Limitations

There are quite significant considerations that may have limited our findings. Our eligibility criteria requiring the HASC professions to have both a provincial regulatory mandate and a pan-Canadian accreditor limited the number of professions included in this survey. The premise was that a regulatory mandate that implements a single set of accreditation standards issued by a single organization would provide consistency in IPE accreditation across Canada for any given profession. Further, such an alignment implies the value given to accreditation in general. That being stated, exclusion from this research should not be perceived as judgement on a profession’s IPE involvement or their value in the HASC academic systems.

Further, the data were collected with a voluntary self-administered online survey and the low response rate is not a surprise. The low response rates may have been impacted by a lack of clarity regarding the individuals responsible for IPE accreditation for each profession. Additionally, the information requested may be regarded by many academic institutions as quite sensitive; accreditation is vital to ongoing functioning and there may be perceived risks associated with disclosures. There are also the usual concerns with self-report, especially in relation to compliance with IPE-relevant standards. Other factors that may have influenced the response rates include the distribution/availability of accreditation documents within academic programs, administrative load on professional programs during the COVID-19 pandemic, format of accreditation documents for ease of ‘cutting and pasting’ responses to survey questions, turnover in IPE leads among academic programs, and IPE leads’ awareness or lack thereof of the CIHC and the AIPHE projects [8, 9]. Lastly, interest in accreditation might be linked to actual participation in IPE initiatives, which we did not measure and may have biased our findings. IPE-rich educational environments, as some academic institutions might be, may heighten the importance given to IPE-relevant accreditation standards and the incentive to respond to this survey.

## Conclusions

Accreditation standards provide a strong incentive to promote and harmonize IPE. In follow-up of the AIPHE projects [8, 9], the findings of this study indicate that HASC professions’ accrediting organizations have since incorporated IPE-relevant standards using varying terminologies and goals, while their respective academic programs are providing the evidence to meet a majority of them. Promising trends were identified through the exemplars collected in this study, particularly in the *Educational Program* domain. More attention is needed to address the challenges raised by HASC academic programs in meeting IPE-relevant accreditation standards and to share best practices both within and across pan-Canadian accrediting organizations. Similarly, more research is needed to evaluate whether these standards enrich student’s IPE experiences, translate to a change in students’ attitudes, beliefs, and behaviors, and promote an interprofessional, collaboration-ready HASC workforce across Canada. Lastly, the array of exemplars described in this paper may be of relevance in advancing IPE implementation and accreditation across Canada and perhaps, more importantly, in countries where these processes are yet emerging.

## Supplementary Information

**Additional file 1.** IPE-relevant text, by profession.

## Data Availability

The datasets supporting the conclusions of this article are available from the corresponding author upon reasonable request.

## Abbreviations

ACOE: Accreditation Council on Optometric Education
AIPHE: Accreditation of Interprofessional Health Education
CACMS: Committee on Accreditation of Canadian Medical Schools
CAD: Commission on Accreditation for Denturism
CAIPE: Centre for the Advancement of Interprofessional Education
CanRAC: Canadian Residency Accreditation Consortium
CAOT: Canadian Association of Occupational Therapists
CASN: Canadian Association of Schools of Nursing
CASWE: Canadian Association for Social Work Education
CCAPP: Canadian Council for Accreditation of Pharmacy Programs
CDAC: Commission on Dental Accreditation of Canada
CFCREAB: Canadian Federation of Chiropractic Regulatory and Education Accrediting Boards
CFPC: College of Family Physicians of Canada
CIHC: Canadian Interprofessional Health Collaborative
CPA: Canadian Psychological Association
EHPIC: Educating Health Professionals in Interprofessional Care
HASC: Health and social care
HiAP: Health in all Policies
HREB: Health Research Ethics Board
HSO: Health Services Organization
HSSA: Health Sciences Students Association
IECPCP: Interprofessional Education for Collaborative Patient-Centred Practice
IPCP: Interprofessional collaborative practice
IPE: Interprofessional education
NHWA: National Health Workforce Accounts
PDEP: Partnership for Dietetic Education and Practice
PEAC: Physiotherapy Education Accreditation Canada
WHO: World Health Organization

## Footnotes

1 In this study, *accountable* text was defined as “a directive or requirement aimed at ensuring a specific IPE or IPCP learner outcome that the accrediting body could reasonably hold the college or school accountable to, based on the statement itself or the context within which it occurred” ([38], p. 125).

2 The three Canadian territories were excluded as there are various pathways to licensure as well as inconsistent regulatory processes in these jurisdictions.

3 According to Barr and Brewer [39], practice-based IPE can be defined as “interprofessional learning in dedicated interprofessional team-based placements providing planned interprofessional interventions with clients” (p. 199).
