# Correction: Accreditation as a driver of interprofessional education: the Canadian experience

**DOI:** 10.1186/s12960-022-00775-4

**Published:** 2022-11-04

**Authors:** Mohammad B. Azzam, Marie-Andrée Girard, Cynthia Andrews, Hope Bilinski, Denise M. Connelly, John H. V. Gilbert, Christie Newton, Ruby E. Grymonpre

**Affiliations:** 1 Curriculum Studies and Studies in Applied Linguistics, Faculty of Education, Western University, London, ON Canada; 2 Anesthesiology and Pain Medicine Department, Faculty of Medicine, University of Montreal, Montreal, QC Canada; 3 Health Hub: Politics, Organizations and Law, Montreal, QC Canada; 4 Department of Dental Clinical Sciences, Faculty of Dentistry, Dalhousie University, Halifax, NS Canada; 5 College of Nursing, University of Saskatchewan, Saskatoon, SK Canada; 6 School of Physical Therapy, Faculty of Health Sciences, Western University, London, ON Canada; 7 College of Health Disciplines, University of British Columbia, Vancouver, BC Canada; 8 Department of Family Practice, Faculty of Medicine, University of British Columbia, Vancouver, BC Canada; 9 College of Pharmacy, Rady Faculty of Health Sciences, University of Manitoba, Winnipeg, MB Canada

## Correction: Human Resources for Health (2022) 20:65 https://doi.org/10.1186/s12960-022-00759-4

Following publication of the original article [1], the authors would like to correct reference 33 and the name of Health Standards Organization.

The incorrect name is: Health Services Organization.

The correct name is: Health Standards Organization.

The incorrect reference is:

Health Services Organization. CAN/HSO 1003:2021 Clinical governance standard—public review. Health Services Organization; 2010. https://healthstandards.org/public-reviews/clinical-governance/.

The correct reference is:

Health Standards Organization. CAN/HSO 1003:2021 Clinical governance standard—public review. Health Services Organization; 2010. https://healthstandards.org/public-reviews/clinical-governance/.

The original article [1] has been corrected.
