# Supplementary material for: Accreditation as a driver of interprofessional education: the Canadian experience
Source: Hum Resour Health. 2022 Aug 26;20:65. doi: 10.1186/s12960-022-00759-4 (PMC9412779; doi:10.1186/s12960-022-00759-4)
Supplement: Supplementary file 1 — Additional file 1. IPE-relevant text, by profession. [file 12960_2022_759_MOESM1_ESM.docx]

| **Additional File 1. IPE-Relevant Text, by Profession** | |
| --- | --- |
| Given the two eligibility criteria for inclusion in this study, only 12 health and social care professions were eligible to participate in this study: chiropractic, dentistry, denturism, dietetics, medicine, nursing (registered), occupational therapy, optometry, pharmacy, physical therapy, psychology, and social work. In preparation for survey development, the accreditation standards documents for these professions were retrieved and reviewed; the Commission on Accreditation for Denturism (CAD) did not respond to our request for their accreditation standards document; therefore, denturism was excluded from this study. Similarly, optometry was further excluded after review of the Accreditation Council on Optometric Education (ACOE)’s accreditation standards document (2014) revealed that their accreditation standards made no reference to interprofessional education or interprofessional collaborative practice. Please note that the six professions involved in the Accreditation of Interprofessional Health Education (AIPHE) projects (2010, 2011) are highlighted in green; the other four professions are highlighted in grey. | |
|  | |
| **CHIROPRACTIC** |  |
|  | |
| 1. The offerings must include … professional practice ethics and interprofessional collaboration (p. 33). | |
|  | |
| 1. Upon completing the course of study, each student will demonstrate … [that they can]: work collaboratively on an interprofessional basis for patient referral and/or management (p. 35). | |
|  | |
| 1. The program must provide evidence that demonstrates that the degree candidates, as a condition of graduation, have: engaged in collaborative activity with other health care and social care providers regarding the care of at least five (5) different patients (p. 41). | |
|  | |
| 1. The student must demonstrate the ability to: recognize the clinical indications for referral to or collaborative care with appropriate mental health professionals, agencies or programmes (p. 48). | |
|  | |
| 1. The student must demonstrate the ability to: identify personal and/or professional care limitations and recognize the need for patient referral and/or collaborative care (p. 52). | |
|  | |
| 1. The student must demonstrate the ability to: identify practices that foster collaboration with other health and social care providers (p. 53). | |
|  | |
| 1. The student must demonstrate the ability to: work collaboratively on an inter or intra professional basis for patient referral and or management as clinically indicated (p. 54). | |
|  | |
| 1. The student must demonstrate the ability to: initiate referral or collaborative care when appropriate to the needs of the patient (p. 54). | |
|  | |
| 1. The student must demonstrate the ability to: recognize the need to monitor the patient’s response to care and modify the care plan, consult with, or refer to another health care provider (p. 57). | |
|  | |
| 1. The student must demonstrate the ability to: recognize and respond to patient concerns and apprehension that may result from proposed changes in a care plan or the need for referral or collaborative care (p. 57) | |
|  | |
| 1. Interpersonal skills should be assessed by reviewing performance in collaboration with staff, members of the patient care team, and consultations with doctors of chiropractic and other health care providers as appropriate (p. 66). | |
|  | |
| **DENTISTRY** |  |
|  | |
| 1. The program must have competencies that describe the graduate of the program. Program-specific competencies must be consistent with the ‘*Competencies for a Beginning Dental Practitioner in Canada’* (p. 12). | |
|  | |
| 1. Describe the program’s relationships with other health sciences educational programs that permit students to develop interprofessional working relationships, as appropriate, with other programs and students (p. 29). | |
|  | |
| 1. The program must have a functional relationship with at least one (1) hospital with a dental service approved by CDAC. This relationship must afford each student the opportunity to participate in the management of a patients’ health and observe working relationships with other health professionals in a hospital (p. 29). | |
|  | |
| 1. Describe the opportunities in place that permit each student to participate in the management of a patients’ health and observe working relationships with other health professionals in a hospital. Attach as an appendix the schedule for these activities (p. 30). | |
|  | |
| 1. Students should be exposed to the principles of interprofessional collaboration for the provision of patient care (p. 30). | |
|  | |
| 1. Identify students’ interprofessional collaborative experiences within the program (p. 30). | |
|  | |
| **DIETETICS** |  |
|  | |
| 1. The curriculum is student/intern centred and based on achieving the Integrated Competencies for Dietetic Education and Practice (ICDEP) (p. 9). | |
|  | |
| 1. The curriculum sequencing is appropriate for progressive student/intern learning. Flow of learning builds in complexity with reinforcement as required to achieve the ICDEP (p. 9). | |
|  | |
| 1. Mapping of the curriculum demonstrating where and how the ICDEP are incorporated into components of the curriculum (p. 9). | |
|  | |
| 1. Course outlines/outcomes and/or practicum/ internship rotations objectives showing a link to the ICDEP (e.g., ICDEP Performance Indictors and Foundational Knowledge) (p. 9). | |
|  | |
| 1. Student/intern tasks during all stages of learning must contribute to meeting ICDEPs in a meaningful way and must be at an appropriate level of complexity (p. 10). | |
|  | |
| 1. A description of learning activities that demonstrate a relationship to the ICDEP (e.g., Academic: Foundational Knowledge cognitive levels 1-3 achieved with course learning activities; Practicum: performance rating scale) (p. 10). | |
|  | |
| 1. The curriculum provides opportunities to develop interprofessional practice skills (p. 10). | |
|  | |
| 1. Demonstration of learning activities within the curriculum that build interprofessional practice skills (p. 10). | |
|  | |
| 1. Preceptors are academically and experientially qualified for their role in assisting interns to achieve the ICDEP (p. 13). | |
|  | |

| **NURSING (REGISTERED)** |  |
| --- | --- |
|  | |
| 1. Partnerships refer to collaborations that support the achievement of the unit’s strategic goals, collaborations among educational units to deliver a collaborative nursing education program, and formal agreements with health service organizations, community-based agencies, members of other professions, and other relevant groups to provide professional and interprofessional learning opportunities for students (p. 12). | |
|  | |
| 1. Faculty are supported in providing interprofessional education and opportunities for intersectoral collaboration (p. 16). | |
|  | |
| 1. Practice placement sites provide learning opportunities that effectively help learners attain the outcomes of the nursing education program(s) and facilitate intra and interprofessional collaboration (p. 18). | |
|  | |
| 1. The curriculum provides learning experiences related to primary health care … and end-of-life care, across the life span of individuals, families, groups, communities, and populations; promotes interprofessional practice, and addresses regulatory entry-to-practice competencies (p. 23). | |
|  | |
| 1. The program provides opportunities for learners to develop knowledge, skills, and attitudes in using relevant information, communication technology, critical thinking, and clinical reasoning, in the delivery of collaborative client-centered care (p. 25). | |
|  | |
| 1. The program provides opportunities for learners to develop the knowledge, skills, and attitudes to provide safe, ethical, and client-centred care as a member of the interprofessional team (p. 26). | |
|  | |
| 1. The program provides opportunities for students to develop functional working relationships, including intra/interprofessional and intersectoral collaboration (p. 26). | |
|  | |
| **MEDICINE (FAMILY MEDICINE)** |  |
|  | |
| 1. The residency program uses a comprehensive curriculum plan, which is specific to the discipline, and addresses all the CanMEDS/CanMEDS-FM Roles (p. 14). | |
|  | |
| 1. The curriculum plan addresses expert instruction and experiential learning opportunities for all the CanMEDS-FM Roles, with a variety of learning activities (p. 14). | |
|  | |
| 1. Residents’ clinical responsibilities are assigned in a way that supports the progressive acquisition of competencies and/or objectives, as outlined in the CanMEDS/CanMEDS-FM roles (p. 14). | |
|  | |
| 1. Resident training takes place in functionally inter- and intra-professional learning environments that prepare residents for collaborative practice (p. 17). | |
|  | |
| **MEDICINE (SPECIALTY MEDICINE)** |  |
|  | |
| 1. The competencies and/or objectives address each of the Roles in the CanMEDS/CanMEDS-FM Framework specific to the discipline (p. 8). | |
|  | |
| 1. The residency program uses a comprehensive curriculum plan, which is specific to the discipline, and addresses all the CanMEDS/CanMEDS-FM Roles (p. 9). | |
|  | |
| 1. The curriculum plan addresses expert instruction and experiential learning opportunities for each of the CanMEDS/CanMEDS-FM Roles with a variety of suitable learning activities (p. 9). | |
|  | |
| 1. Residents’ clinical responsibilities are assigned in a way that supports the progressive acquisition of competencies and/or objectives, as outlined in the CanMEDS/CanMEDS-FM Roles (p. 9). | |
|  | |
| 1. Residents’ clinical responsibilities, including on-call duties, provide opportunities for progressive experiential learning, in accordance with all CanMEDS/CanMEDS-FM Roles (p. 9). | |
|  | |
| 1. The system of assessment meets the requirements within specific standards for the discipline, including achievement of competencies in all CanMEDS or CFPC evaluation objectives, as applicable (p. 10). | |
|  | |
| 1. Resident training takes place in functionally inter- and intra-professional learning environments that prepare residents for collaborative practice (p. 12). | |
|  | |
| **MEDICINE (UNDERGRADUATE MEDICINE)** |  |
|  | |
| 1. The faculty of a medical school ensure that the core curriculum prepares medical students to function collaboratively on health care teams that include health professionals from other disciplines as they provide coordinated services to patients. These required curricular experiences include practitioners and/or students from the other health professions (p. 13). | |
|  | |
| **OCCUPATIONAL THERAPY** |  |
|  | |
| 1. A report that documents and critically reflects upon the inclusive educational methods (including fieldwork education) and their consistency with the educational and professional conceptual frameworks including interprofessional education and practice (p. 17). | |
|  | |
| 1. Academic and fieldwork education components incorporate interprofessional education (p. 19). | |
|  | |
| 1. A report that documents the IPE activities and experiences integrated in the occupational therapy program. The report should describe the program offerings, and include considerations of space, human and learning resources required to deliver IPE (p. 19). | |
|  | |
| **PHARMACY** |  |
|  | |
| 1. Students demonstrate practice-readiness that enables them to provide patient care as a collaborative member of a care team before starting culminating direct patient care required practice experiences (p. 7). | |
|  | |
| 1. The professional degree program in pharmacy has a minimum of four academic years, or the equivalent number of hours or credits, including a series of core courses, practice experiences and interprofessional experiences that support educational outcomes (p. 9). | |
|  | |
| 1. The Faculty ensures that the professional program includes diversity of required and elective courses, practice experiences, and intra- and inter-professional educational experiences that incorporate different levels of patient acuity, and an organized progression in the level of expected performance that supports growth in students’ capabilities to meet educational outcomes (p. 10). | |
|  | |
| 1. The curriculum addresses outcomes and competencies to develop graduates that are capable of carrying out care provider, communicator, collaborator, leader manager, health advocate, scholar and professional roles (p. 10). | |
|  | |
| 1. The curriculum includes required intra- and interprofessional learning experiences, offered throughout the professional program, to enable a graduate to provide patient care as a collaborative member of a care team (p. 12). | |
|  | |
| 1. Experiences address content to develop the expected competencies for intra- and interprofessional care and collaborative practice. Experiences are integrated throughout the professional program (p. 12). | |
|  | |
| 1. The University has integrated and endorsed the concept of interprofessional education and collaboration in practice (p. 16). | |
|  | |
| 1. The University enables relationships that support interprofessional learning (p. 16). | |
|  | |
| 1. Organizational structures and processes are in place to support interprofessional education (p. 16). | |
|  | |
| 1. Interprofessional education is recognized as a valuable teaching responsibility within the academic health sciences (p. 17). | |
|  | |
| 1. Interprofessional education and collaborative practice is embedded in Faculty policy and/or strategic plans (p. 20). | |
|  | |
| 1. The policy and/or strategic plan includes the evaluation of interprofessional education (p. 20). | |
|  | |
| 1. The Faculty works collaboratively with practice sites and other health professions programs to make intra- and/or inter-professional care/collaborative practice environments available to student pharmacists (p. 27). | |
|  | |
| **PHYSIOTHERAPY** |  |
|  | |
| 1. The program prepares students to use effective communication to develop professional relationships with clients, families, team members, care providers, and other stakeholders (p. 29). | |
|  | |
| 1. The program prepares students for collaborative practice to support quality client-centred care (p. 30). | |
|  | |
| 1. Establish and maintain interprofessional relationships, which foster effective collaborative practice (p. 30). | |
|  | |
| 1. The program prepares students to: demonstrate an understanding of and respect the roles, responsibilities, and differing perspectives of team members including clients (p. 30). | |
|  | |
| 1. The program prepares students to: consult and share relevant information with clients, other health professionals, and all relevant individuals or groups in a timely manner (p. 30). | |
|  | |
| 1. The program prepares students to: promote active and informed shared decision making (p. 30). | |
|  | |
| 1. The program prepares students to: foster collaboration with relevant others (p. 30). | |
|  | |
| 1. Prevent, manage, and resolve conflict related to client-centred care (p. 30). | |
|  | |
| 1. The program prepares students to: demonstrate a respectful attitude towards colleagues and members of an interprofessional team, including clients (p. 30). | |
|  | |
| 1. The program prepares students to: identify the issues that may contribute to the development of conflict between the physiotherapist and client or among team members (p. 30). | |
|  | |
| 1. The program prepares students to: address conflicts in an appropriate and timely manner (p. 30). | |
|  | |
| 1. Work collaboratively to identify, respond to, and promote the health needs and concerns of clients (p. 31). | |
|  | |
| 1. The program prepares students to: collaborate with clients and other care providers to understand, identify, and promote the health and physiotherapy needs and concerns of clients (p. 31). | |
|  | |
| 1. The program prepares students to: understand the limits and opportunities in the practice setting to address health issues, and work collaboratively to develop strategies to optimize client care (p. 31). | |
|  | |
| **PSYCHOLOGY** |  |
|  | |
| 1. Intervention in clinical neuropsychology includes: consultation to the community and other institutions (e.g., schools, other health or residential care facilities) and inter-professional teams about the cognitive and psychological functioning and needs of patients with neurological disorders (p. 35). | |
|  | |
| **SOCIAL WORK** |  |
|  | |
| 1. Social work students are prepared for interprofessional practice, community collaboration and team work (p.12). | |
